# A Framework and Improvements of the Korea Cloud Services Certification System

**DOI:** 10.1155/2015/918075

**Published:** 2015-06-01

**Authors:** Hangoo Jeon, Kwang-Kyu Seo

**Affiliations:** ^1^Department of Management Engineering, Graduate School, Sangmyung University, Seoul 110-743, Republic of Korea; ^2^Department of Management Engineering, Sangmyung University, Cheonan 330-720, Republic of Korea

## Abstract

Cloud computing service is an evolving paradigm that affects a large part of the ICT industry and provides new opportunities for ICT service providers such as the deployment of new business models and the realization of economies of scale by increasing efficiency of resource utilization. However, despite benefits of cloud services, there are some obstacles to adopt such as lack of assessing and comparing the service quality of cloud services regarding availability, security, and reliability. In order to adopt the successful cloud service and activate it, it is necessary to establish the cloud service certification system to ensure service quality and performance of cloud services. This paper proposes a framework and improvements of the Korea certification system of cloud service. In order to develop it, the critical issues related to service quality, performance, and certification of cloud service are identified and the systematic framework for the certification system of cloud services and service provider domains are developed. Improvements of the developed Korea certification system of cloud services are also proposed.

## 1. Introduction

Cloud service is a technology which makes computing resources available to any device at anytime and anywhere if accessible via Internet. The opening of cloud service era means a change from the existing hardware, platform, or software oriented to the service oriented business model. This would bring about great change to the related companies as well as the lives of those who use services in every area including public institutions and individuals [1]. Cloud computing services are an evolving paradigm that affects a large part of the ICT industry and provides new opportunities for ICT service providers, such as the adoption of new business models and the realization of economies of scale by increasing efficiency of resource utilization [2]. In addition, users of cloud services have a lot of benefits and advantages such as a high degree of flexibility and low upfront capital investments [3]. However, despite advantages of cloud services, there are some obstacles to adopt such as lack of assessing and comparing the service quality of cloud services regarding availability, security, and reliability. In addition, small and medium companies lack appropriate, qualified, and trustworthy information and methodology to evaluate and compare cloud services with regard to advantages and associated risks [4].

There are a few studies regarding cloud service certification. The Federal Risk and Authorization Program (FedRAMP) is a risk management program that provides a standardized approach for assessing and monitoring the security of cloud products and services [5, 6]. The program is intended to facilitate the adoption of cloud computing services among federal agencies by providing cloud service providers (CSPs) with a single accreditation that could be used by all agencies. Certifications are based on a unified risk management process that includes security requirements agreed upon by the federal departments and agencies [7]. Kim et al. suggested core evaluation criteria and added evaluation criteria which removed the redundancy of the security controls from existing ISMS for Korean cloud computing through a comparative analysis between domestic and foreign security controls of cloud certification scheme and guidelines and information security management system [8]. Kou surveyed the existing information assurance scheme [9], especially FedRAMP, and proposed security-enhanced cloud service evaluation and certification scheme. Schneider et al.'s study delineated and structured cloud service certification knowledge by developing a taxonomy for criteria to be assessed in a cloud service certification [10]. Sunyaev and Schneider presented the necessity and issues about cloud services certification and discussed how to address the lack of transparency, trust, and acceptance in cloud services. But they did not propose a specific cloud services certification [4].

The previous studies regarding cloud service certification focused on security issue of cloud service and overview of it. Most of the current cloud computing related studies have focused on the cloud technology itself. However, it is also urgent to understand the issues in the business aspect surrounding the cloud service. To successfully provide and activate cloud services, we need to establish a certification system to assure service quality of cloud services regarding availability, security, reliability, and so on.

This paper proposes the framework of the Korea cloud service certification system to guarantee quality, performance, safety, reliability, and so forth of cloud services and to activate cloud industry in Korea. It assesses and certifies service quality, stability and security, and so forth of cloud services and providers to promote the expansion of the market demand for cloud service. In addition, improvements of the developed framework of the Korea certification system of cloud services are proposed.

There is currently no way to verify the safety and reliability when a public or private organization implements cloud service in Korea. Therefore, the Korean Cloud Service Certification Committee has been created and its members consist of experts from governmental, academic, industrial organization. The committee was led by a Korean government agency (Ministry of Science, ICT and Future Planning) to develop Korean cloud service certification system. This study introduced the Korean cloud service certification framework that has been developed by the Korean Cloud Service Certification Committee. The Korean cloud service certification system is applied to domestic and foreign cloud services being provided in Korea for their certification. Currently, it is being implemented for a private level certification with a future plan to expand it as a government level certification such as FedRAMP.

**Figure 1:**
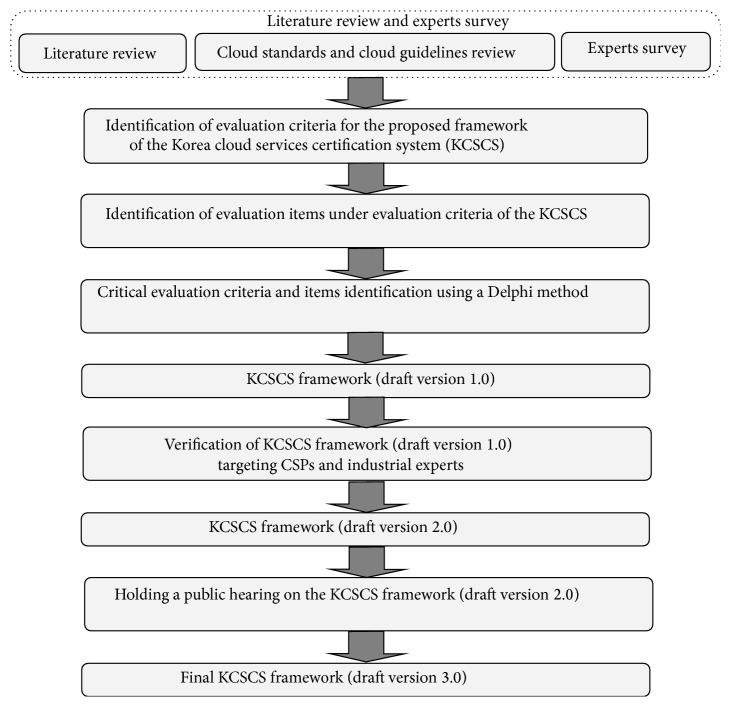
The development procedures of the Korea cloud service certification system.

Figure 1 shows the development procedures of the Korea cloud service certification system. As shown in Figure 1, specific evaluation items were deduced according to the higher assessment categories of the framework by using Delphi method. Opinions of cloud service providers and related industry experts were collected for verification of the proposed framework. In particular, industrial experts consist of not only domestic CSPs such as KT, SKT, LG U+, and so forth but also tenants and resellers of global cloud services provided by amazon, google, Microsoft, and so forth in order to verify the applicability of the proposed Korean cloud service certification system in domestic and global cloud services. The proposed certification system is reviewed and analyzed by both of them. All of domestic industrial experts and tenants and resellers of global cloud services agreed upon and confirmed the feasibility and validity of the proposed Korean cloud service certification system. Finally a public hearing was held to evaluate adequacy and suitability of the framework of Korea cloud service certification system.

The certification system proposed in this study is applicable for cloud services and providers currently being active in Korea and the term “in Korea” is used because it is the only cloud service certification system in Korea.  Table 1 shows that various cloud services are being serviced in Korea but there are few PaaS. Domestic and global cloud services in Table 1 are candidates to obtain the proposed Korea cloud service certification system.

The remainder of the paper is organized as follows. In the next section, the problems of cloud services are presented that are user protection problems in cloud services and provider support and protection problems of cloud services. A framework of the Korea cloud service certification system is described in Section 3. The evaluation criteria and items of it are provided and the evaluation method is also proposed.  Section 4 presents improvements of the developed Korea cloud service certification system. General conclusions and future works are drawn in Section 5.

## 2. Problems of Cloud Services

Cloud service, which is an alternative for companies to reduce costs in the economic recession, has the advantage that could obtain the service with a minimum initial cost, its service implementation is faster than the existing methods, and the maintenance cost is cheaper. Nevertheless, cloud service has problems such as security and availability, and they are as follows.

- Security problem: whenever a new technology is introduced in the IT market, the most problematic matter is its security. Cloud service could not also avoid such a problem. Even though cloud providers use specialized technologies (e.g., encryption), processes (e.g., verifiability), verification standards (e.g., PCI and ISO 27001), and so forth to solve the security problem, it is less likely to use cloud service for the vitally important data and processes at present [11].
- Availability problem: companies necessarily need to use Internet for receiving cloud service. However, they could not receive the service if Internet is not easy to access or the service provider's system fails. To expand cloud service, it should make further efforts on implementing the system of basically providing the service at anytime and anywhere [12].
- Performance problem: Internet speed and bandwidth are closely related with cloud service's performance. How fast and how many data could be transmitted also become a key index of evaluating the service's performance [13].

### 2.1. User Protection Problems in Cloud Services

Despite many advantages of cloud service, users feel an anxiety in various aspects. In particular, there is great concern about how much users could be protected if a problem arises in cloud service [14]. Recently, users' anxiety grows because of Google Gmail's access failure, Twitter's internal data leakage, Amazon's service interruption, and so forth, so there is a growing demand for securing stability and reliability of cloud computing service, and the following points should be considered as the user protection problem in cloud service [15].

- Provider's bankruptcy: if a provider is bankrupted, cloud service's users could not but suffer an enormous loss. When the provider is bankrupted, the existing service users could not use the service so stably that their business carried out with the service until now is stopped. In addition, if the data is damaged or lost, the information or data stored in the cloud service until now would be lost. After the service provider's bankruptcy, even if another service provider could be found, there might be problems of whether the existing provided service could be equally offered or whether the systems between the companies are compatible.
- Service interruption and failure: if cloud computing service is interrupted or failed, users also suffer similar damage to the case of service provider's bankruptcy. In particular, for cloud service, because its respective services and computers are connected via networks like Internet, it is vulnerable to the security incident such as virus infection and hacking, so there is a chance that the service fails at any time. Furthermore, because cloud computing service is provided as a form of storing users' information in the service provider's server, the scale of information loss or damage due to the service failure is much larger than other Internet-based services. To prevent it, it is needed to clearly define the scope of compensation for damages caused by service failure in the regulation of the cloud service provider's user agreement. In the exemption provisions for compensation, it considers not only the provider's position unilaterally but also the service users' position together, so it should be able to relieve the service users' anxiety when using the service.
- Protecting users' information: the core technology of cloud service is virtualization. Therefore, it is difficult to find the positions of data stored by users, vulnerable to outside attacks and at great risk of leaking sensitive personal information if administrators misuse or abuse their authority.
- Strengthening the platform's independence: the platform's independence means that the platform could carry out works regardless of the operating system or terminal. In cloud service, strengthening the platform's independence is more important than anything else. If cloud service provider is unexpectedly bankrupted or abandons his business, it is inevitable that the existing users, who use the relevant service, change into another service. Then, if the platform is independent, users could easily move to other services without any transition cost. Therefore, it could be said that strengthening the platform's independence in cloud service is an extension of the institutional strategy which could protect users' right and information.

### 2.2. Provider Support and Protection Problems of Cloud Services

Judging from the provider side, user's anxiety about cloud service could be the burden of the provider. Users' anxiety looked at from the providers' side could be divided into security and safety of user information. In the providers' position, they should keep the security of user information and the safety for preventing the user information loss, but the measures to it are currently at an insignificant level [16].

- Securing the business security: one of the cloud computing problems most frequently pointed out is the service's security. As mentioned in the user's aspect, users might be anxious about the security because of the outside attacks such as hacking and the administrator's abuse problem. The users' anxiety could be developed instantly to the providers' anxiety, and if users suffer damage due to the security problem while carrying out the service in the condition of not securing the security for users' information, providers should also prepare the compensation for the damage. However, the scope and amount of compensation for users are not also exact at present, so it is expected that there would be lots of problems.
- Therefore, providers should establish sufficient security measures and make regulations to assure the security factors suitable for cloud service in providing the service. Furthermore, it is needed to add the security items for the virtualization server, which are technologies that newly emerged due to the cloud computing, or also the items defining data recovery or backup, and so forth, which is handled carelessly in the existing system, to establish a new certification system for cloud service.
- Service stabilization through mutual compatibility: the compatibility between providers could stabilize the cloud service. Looking from the short-term view, the compatibility might integrate respective providers' characteristics to reduce their profits. Therefore, the compatibility between providers has not been highly achieved in the current cloud service industry. In addition, the legislation prescribing is also insufficient so that many problems are caused in stabilizing the service. First of all, only if respective providers bring out individual business items after every provider lowers the accident rate through mutual compatibility and standardization and consistently organizes the concept to widen the overall market size, the profitability could be secured.
- Strengthening the provider's capability: looking from the provider's side, the direct support such as manpower support, tax exemption, institutional complement, and deregulation in the government or private sector is also one of the important factors. But there is no support system for the companies using the cloud service because it is not long after introducing the cloud service technology to actually carry out the service. Firstly, the company carrying out cloud service is defined vaguely, and the support range is not determined. Because of that, for the company providing cloud service, its support target and scale are not so correct which is difficult to support, so it is needed to prepare measures to it.
- Certification system: the certification system is a method to guarantee the effect of a product and technology so that users could use it reliably. In the current situation of being not long after cloud service was introduced, the certification system could reduce the anxiety of most people who did not know cloud service about new technologies and services. By doing so, providers would be able to develop technologies and services more drastically with users' confidence to activate the cloud service industry [10].

Service Level Agreements (SLAs) are agreements signed between a service provider and another party like a service consumer, broker agent, or monitoring agent. Because cloud computing is recent technology providing many services for critical business applications, the need for reliable and flexible mechanisms to manage online contracts is very important [17]. Therefore it is necessary to develop SLAs among cloud providers and cloud consumers. In Korea, the guideline of Cloud Service Level Agreement was developed by Korea Communications Commission in October 2011 [18]. This guideline includes the detailed agreement conditions: (1) service availability, (2) data backup, restore, and security, (3) customer support, and (4) charges for breach of contract. It additionally contains contract condition, service security, service scalability, service level, report provision of service level, and so on.

As mentioned above, it is necessary to prepare cloud service certification system for all cloud service users and providers. Cloud service certification system could solve many problems which might arise in cloud service, and it could be said that it is a core element for providing reliable and safe services between users and providers. Therefore, this study proposes the framework of the Korea cloud service certification system prepared for solving these problems.

## 3. A Framework of the Korea Cloud Service Certification System

### 3.1. Korea Cloud Service Certification System

**Figure 2:**
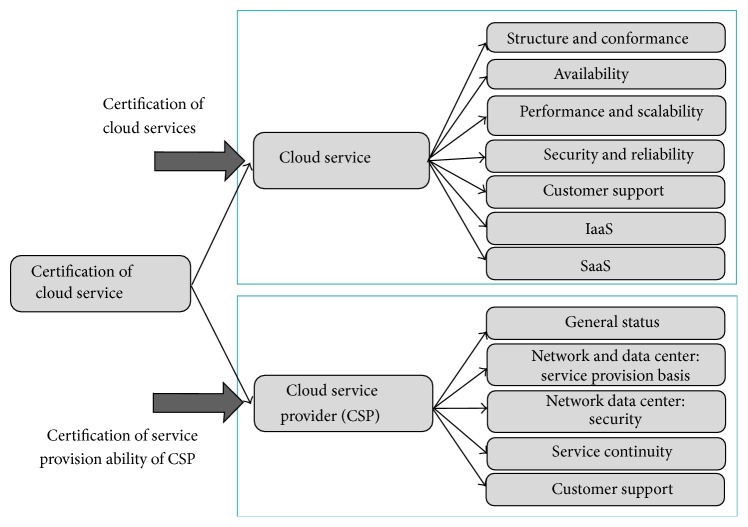
Certification scope and evaluation criteria for cloud services.

Korea cloud service certification system evaluates two areas of “cloud service” and “cloud service provider.” For cloud service, five evaluation criteria such as the structure examination and conformance, availability, performance and scalability, security and reliability, and customer support are comprehensively assessed, but items for each service (IaaS and SaaS) are added to differentiate the evaluation standard. As mentioned before, there are few PaaS in Korea, so we do not include PaaS certification in the proposed certification system. For the cloud service provider, five evaluation criteria such as general status, network/data center (service provision basis and security), service continuity, and customer support are comprehensively evaluated. The cloud service certification scope and evaluation criteria are shown in Figure 2.

Existing ISPs are excluded from certification and propriety of certification is reviewed in Section 3.1.1 (1) Structure and conformance. In addition, simple streaming services or Web-hard services are also excluded from certification.

The more detailed description of evaluation criteria and items is presented as follows. In this section, we only explain the detailed description of availability as an example among evaluation items because of the extensive amount of information of all of them.

#### 3.1.1. Common Criteria of Cloud Service

*(1) Structure and Conformance*. These criteria evaluate cloud service structure's usefulness and conformance such as the cloud service's functions and logical and physical structures. The detailed evaluation items of them are shown in Table 2. These evaluation items were based on standard for SW quality certification (Numbers 5, 6, and 17), ASPIC ASP Delivery Model of USA.

*(2) Availability*. This criterion evaluates whether cloud service is provided to multiple users via Internet. The detailed evaluation items of it are shown in Table 3. These evaluation items were based on ASPIC ASP Delivery Model of USA and we modified it considering cloud service characteristics.

Table 4 shows the evaluation contents and verification items of availability. As shown in Table 3, contents of evaluation items, contents of specific evaluation items, and verification method are explained in detail. As mentioned above, availability as an example among evaluation items is only explained in the paper because of their extensive amount of information.

*(3) Performance and Scalability*. These criteria evaluate whether the service provider works for keeping and improving the cloud service's performance, stability, and scalability. The detailed evaluation items of them are shown in Table 5. These evaluation items were based on standard for SW quality certification (number 10), ASPIC ASP Delivery Model of USA.

*(4) Security and Reliability*. These criteria evaluate the security policy, technology, and so forth, to protect cloud service user's information. The detailed evaluation items of them are shown in Table 6. These evaluation items were based on guides for information protection.

#### 3.1.2. Common Criteria of IaaS and SaaS

*(1) Structure and Conformance of IaaS*. These criteria evaluate whether or not cloud service related technologies such as the virtualization, distribution computing, system management, and metering technologies are applied to provide IaaS. The detailed evaluation item of them is shown in Table 7. These evaluation items were based on standard for SW quality certification (numbers 5, 6, and 17), ASPIC ASP Delivery Model of USA.

*(2) Structure and Conformance of SaaS*. These criteria evaluate the application functions, maturity, data compatibility, data interference or not, and so forth. The detailed evaluation items of them are shown in Table 8. These evaluation items were based on standard for SW quality certification (numbers 5, 6, and 17), ASPIC ASP Delivery Model of USA.

*(3) Availability of SaaS*. This criterion evaluates the application's integrity and the function to check accessible time. The detailed evaluation items of it are shown in Table 9. These evaluation items were based on ASPIC ASP Delivery Model of USA and we modified it considering SaaS characteristics.

#### 3.1.3. Evaluation Criteria of Cloud Service Provider

*(1) General Status*. This criterion checks general status such as the cloud service provider's company name, establishment year, representative, organizational, and manpower status, and charging system to evaluate whether or not the management basis is prepared to provide cloud service. The detailed evaluation items of it are shown in Table 10.

*(2) Network and Data Center*—*Service Provision Basis*. These criteria evaluate whether cloud service provider secures and maintains an infrastructure for providing the service such as hardware and software resources, support and cooperation organization, and professionalism of technical manpower. The detailed evaluation items of them are shown in Table 11. These evaluation items were based on standard for security and reliability of IDC facility.

*(3) Network and Data Center*—*Security*. These criteria evaluate whether or not cloud service provider establishes and carries out a security plan in the physical, technical, and administrative aspect to protect users' data, prevent security incidents, and so forth. The detailed evaluation items of them are shown in Table 12. These evaluation items were based on surveillance standard for information system, guides for information protection, and standard for security and reliability of IDC facility.

*(4) Service Continuity*. This criterion evaluates whether or not a technical and administrative action plan is established and carried out to assure the service continuity against the possible cloud service interruption. The detailed evaluation items of it are shown in Table 13. These evaluation items were based on surveillance standard for information system.

*(5) Customer Support*. These criteria and items evaluate whether or not activities are carried out for the cloud service's systematic performance and the customer support such as customer education, quality assurance, and A/S.

The detailed evaluation items of them are shown in Table 14.

### 3.2. Evaluation Method of the Korea Cloud Service Certification System

The evaluation method of the cloud services certification system is described briefly.

Both cloud service and cloud service providers are evaluated and the Likert scales (5 points) are applied to assess evaluation items. Certification may gain one of cloud service and cloud services provider certification or may gain both of them according to the operating types of cloud service. All evaluation items in the two certification areas are essential elements in the Korea cloud service certification system. Therefore, in order to gain cloud services and cloud service provider certification must receive at least a certain score for all required fields. The evaluation method of the current certification system has the same weight of each evaluation criterion and each item even though the importance of them is different. There are two certification grades of cloud service such as cloud service certification and cloud service certification with excellent SLA.

Evaluation items are divided into essential and optional items and Likert scales are not used for essential items. For optional items, the certification evaluation committee members qualitatively evaluate documents submitted. During this process, mean value of assessment results by more than 3 committee members is selected to exclude the dependence on an evaluator.

**Figure 3:**
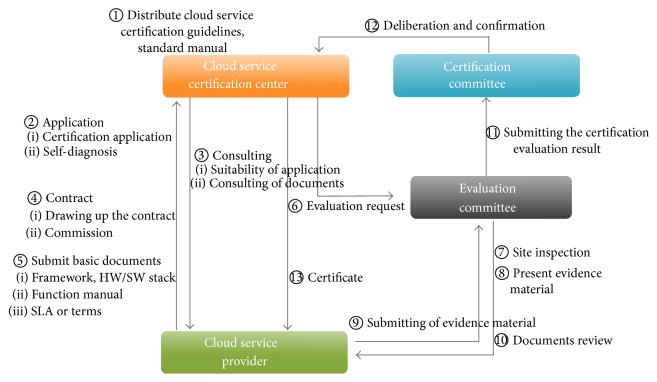
Certification flow of the Korea cloud service certification system.

The detailed certification flow of the Korea cloud service certification system is shown in Figure 3.

### 3.3. Problems of the Developed Korea Cloud Service Certification System

Cloud industry after hearing of the cloud service certification system requests to modify and improve the proposed certification system's complexity and insufficient follow-up support function and so on. The cloud industry's opinion is that there are continuing difficulties of companies such as excessive submission document when examining the certification, accompanying concerns about technology leakage, and time-consuming preparatory period. Accordingly, it offers suggestions such as expanding incentives and introducing the certification grade system.

Therefore, some supports are needed to improve the cloud service's quality competitiveness such as simplifying the certification acquisition procedure and complementing the quality certification consulting function. In addition, a lack of benefits for the certified companies is a main obstacle factor for activating the certification, so it could be said that there is a need to secure effectiveness by strengthening the follow-up support function for the certified companies.

In addition, the security evaluation of the proposed framework should be improved by comparing the other security standards such as ISO 27001, ISMS, and PIMS. The comparison results of security among the proposed Korea cloud service certification system, ISO 27001, ISMS, and PIMS are shown in Table 15.

The proposed framework includes contents on cloud service performance, availability, and security but ISO 27001 and ISMS focus on the security related certification of information system. More details are as follows:

- ISO 27001: a total system established for continuous management and operation by systematically establishing information protection management procedure and process to protect important information asset of organizations;
- ISMS: technical characteristics of organization such as tasks, organization, location, and asset considered;
- PIMS: personal information protection policy and organization throughout the entire process of personal information lifecycle from creation and collection to use, change, and disposal, CCTV installation and management, and technical protective measure of personal information.

Eventually security criteria and items should be more improved and enhanced by incorporating new security evaluation criteria and items of other standards and security evaluation systems and information protection management systems, and so forth, into the proposed framework.

## 4. Improvements of the Korea Cloud Service Certification System

Looking at cloud service certification system's problems derived from the previous section, an improvement method for the Korea cloud service certification system could be suggested as follows.

It is needed to solve difficulties when the cloud companies acquire the certification by subdividing the current cloud service certification system's certification grades for each certification area and simplifying the evaluation indexes centered on necessary evaluation ones. By doing so, the cloud companies' burden of acquiring the certification could be reduced, and it could contribute to spreading the certification system.  Table 16 shows the certification grade improvement method and its content.

In detail, the improvement method is, for cloud service certification, to change the certification grade name into cloud service quality level (levels 1~2) and divide the certification grade level for each certification area (service quality, service basis, and service information security) and, for the excellent SLA cloud service certification, to change the certification grade name into cloud service quality level (levels 3~5) and tighten the screening and evaluation criteria for each grade.

For the specific certification content for each level, first, level 1 is certified if the check items required for the service quality (availability, scalability, and performance) and the service basis (service support) are passed.

Next, level 2 is certified if level 1 and the check items required for the service information security (data management and security) are passed.

Level 3 should satisfy availability above 99.5% and is certified if more than 70% of levels 1~2 general and required check items are passed. And if the evaluation item's availability is more than 99.5%, it could be replaced with the availability analysis result report. However, the required check items should be passed with 100%.

Level 4 should satisfy level 3 conditions with 100% and is certified if the ISMS (information security management system) and ISO 27001 are acquired. The ISMS certificate or ISO 27001 certification document is also required.

Finally, level 5 should satisfy level 4 conditions with 100% and is certified if the user compensation insurance (e.g., liability insurance) is completely prepared. The amount of compensation should not be calculated by the service fee according to the availability but that should be computed by the previous quarter's service sales.

In addition, the weight of evaluation criteria and items is determined by using multi-criteria decision making model (MCDM) such as analytical hierarchy process (AHP) or fuzzy AHP.

## 5. Conclusion

The problem about whether cloud service providers could reflect respective unique characteristics to provide successful services in developing cloud service industry might arise. Cloud service is contracted by the need between an independent service provider and a customer. It should lead to frequent evaluations for the independent and temporary contract, so there is a safety and reliability problem for the cloud service, and to solve this problem and develop domestic cloud service industry, it is needed to establish a safe, reliable, and systematic certification system for the cloud service. In addition, because various types of service providers are included in cloud service business model, it is needed to certify whether respective service providers have proper capability.

There is currently no way to verify the safety and reliability when a public or private organization implements cloud service in Korea. Therefore the Korean Cloud Service Certification Committee has been created to develop Korean cloud service certification system. This study presented the developed Korean cloud service certification framework. The Korean cloud service certification system can be applied to domestic and foreign cloud services being provided in Korea for their certification. A framework to certify entire cloud service has not been found among existing studies and the originality of the cloud service certification system proposed in this study is recognized. There is no existing cloud service certification system in the literature; in other words it is the first cloud service certification system in the literature and it is different from FedRAMP in USA which focuses on only security issues. In the industrial aspect, there is a contribution for revitalizing cloud service and related industries in Korea. The proposed framework is expected to be used not only in Korea but also in other countries and organizations that require the cloud service certification. Therefore this study has the originality and contribution in both academic and industrial aspects.

For the future study, the evaluation criteria and items should be developed to certify the PaaS service, and the weighting method should be also developed according to the importance between the detailed evaluation criteria and items developed. The security criteria and items should be more improved and enhanced. In addition, it is also needed to develop a specific cloud service supervision methodology to evaluate whether the contract between cloud service providers and customers is performed with maintaining a proper quality.

## Figures and Tables

**Table 1:** Domestic and global cloud services in Korea.

Division	IaaS		SaaS	
	Company	Service	Company	Service
Local cloud service firm	KT	U Cloud	Hancom	Net Peace
	SKT	T Cloud	Younglimwon	Younglimwon
	LG U+	U+ Cloud N	Soft Lab	ERP
	CJ Hellovision	Vision Cloud	Duzon	Smart A
	Hyosung ITX	ITX Cloud	Handysoft	Handypia
	Smile Serve	Cloud V	Tilon	L Cloud
	KINX	ixCloud	SKT	Cloud CRM
	Hostway	FlexCloud	Daou	Team Office
	Innogrid	Cloudit		

Global cloud service firm	Amarzon	AWS	Oracle	ERP, HCM
	MS	MS Asure	Google	Google Apps
	Rackspace	Rackspace Server	SAP	HCM
	Dimension Data	Dimension Data	MS	Dynamics ERP

**Table 2:** Evaluation items of structure and conformance.

Evaluation criteria	Evaluation item
Structure and conformance (3 items)	(1) Existence of a specific and detailed manual for users to easily understand cloud service's functions and to be used in the application area
	(2) Existence of a specific and detailed manual for users to easily understand cloud service's logical structure and to be used in the application area
	(3) Existence of a specific and detailed manual for users to easily understand cloud service's physical structure and to be used in the application area

**Table 3:** Evaluation items of availability.

Evaluation criteria	Evaluation item
Availability (3 items)	(1) Whether or not multiple users are supported to simultaneously access cloud service
	(2) Whether or not users are supported to access via various access environments (Web and mobile, etc.)
	(3) Whether or not users are supported to always use cloud service

**Table 4:** Evaluation contents and verification items of availability.

Number (1)	Evaluation purpose	Evaluation content	Number (2)	Evaluation item	Description	Number (3)	Verification item	Method		Essential
								Document	Actual inspection	
1	Availability	Cloud service provider needs to take necessary actions to constantly provide cloud service according to contracted contents.	1.1	Availability policy establishment	For ensuring the conformance of service availability level, cloud service provider needs to establish and document availability policy including present condition on the application of cloud related technologies such as virtualization and physical resource retention/management and system operation/management method. In addition, corresponding policy needs to conform to related legislations, and so forth.	1.1.1	For ensuring cloud service availability level, has the policy been established and documented by defining the items required for management/operation?	O	X	Essential
						1.1.2	Does the availability policy reflect related legislations and include the contents of guidelines provided by the government and cloud service industry?	O	X	Essential
			1.2	Availability goal and result notification	Cloud service provider needs to inform service availability level in advance in documents and prepare related processes periodically to allow users to be aware of availability level.	1.2.1	Is the cloud service availability level being provided to users in advance in documents through basic contract terms and conditions or service level agreement (SLA)?	O	X	Essential
			1.3	Organization and responsibilities	For ensuring the availability level presented to users, cloud service provider needs to appropriately assign organization and personnel and corresponding duties need to be responsibly performed.	1.3.1	For maintaining cloud service availability level, are the organizational roles and responsibilities and correlation with other duties defined and included in availability policy?	O	X	Essential
			1.4	System implementation/ management	For ensuring service availability, cloud service provider needs to compose the system by implementing appropriate cloud technologies/software such as virtualization and distributed computing, and so forth. In addition, monitoring, clustering and maintenance/repair need to be available in case of any system trouble.	1.4.1	Does the cloud system consist of technologies and software needed to provide high availability?	O	O	Essential
						1.4.2	In the case of any cloud service trouble, is there any monitoring technology/system available to detect and identify such trouble in real-time?	O	O	Essential
						1.4.3	Are the core system components composed in clustering to prepare for any cloud service trouble?	O	O	Essential
						1.4.4	Is periodic maintenance/repair being performed to prepare for any cloud service trouble?	O	X	Essential
			1.5	Internal management	Cloud service provider needs to prepare for any service availability limitations to predict/analyze/improve them and retain/manage necessary technical data.	1.5.1	Is the process for analyzing/predicting cloud service availability level available?	O	X	Essential

**Table 5:** Evaluation items of performance and scalability.

Evaluation criteria	Evaluation item
Performance/ scalability (4 items)	(1) Whether or not periodical tests should be used to keep cloud service's performance
	(2) Whether or not periodical tests should be used to keep cloud service's stability
	(3) If cloud service's demand (users, etc.) increases, whether or not there is a method to stably keep the service's performance
	(4) If cloud service's users increase, whether or not that secures the scalability

**Table 6:** Evaluation items of security and reliability.

Evaluation criteria	Evaluation item
Security/ reliability (9 items)	(1) Whether or not the security policy and channel are secured by providing cloud service
	(2) Whether or not the access control is applied to unauthorized users
	(3) Whether or not the integrated authentication process is secured to verify users
	(4) Whether or not cloud service user data's confidentiality and integrity are secured
	(5) Whether or not firewall is introduced to protect cloud service users
	(6) Whether or not a management solution such as monitoring and tracking is introduced by analyzing the log file for preventing and tracking unauthorized users' access
	(7) Whether or not the nonrepudiation function is provided at the messaging level (except the transmission level)
	(8) Whether or not the personal privacy protection function is provided at the messaging level (except the transmission level)
	(9) Existence of the measures to the service and data's failure

**Table 7:** Evaluation items of structure and conformance of IaaS.

Evaluation criteria	Evaluation item
Structure and conformance (1 item)	(1) Whether or not cloud service related technologies such as the virtualization, distributed computing, system management, and mirroring technologies are applied to provide the IaaS service

**Table 8:** Evaluation items of structure and conformance of SaaS.

Evaluation criteria	Evaluation item
Structure and conformance (4 items)	(1) Existence of a specific and detailed manual for users to easily understand and use the application functions
	(2) Existence of a manual for the maturity model (level) of technologies applied to the application
	(3) Whether or not the compatibility is supported when transmitting the SaaS service's data
	(4) SaaS service's data interference or not

**Table 9:** Evaluation items of availability of SaaS.

Evaluation criteria	Evaluation item
Availability (2 items)	(1) Check the SaaS integrity of application
	(2) Check the total use time and accessible time

**Table 10:** Evaluation items of general status.

Evaluation criteria	Evaluation item
General status (3 items)	(1) Verify cloud service provider's identity/potential for providing the service
	(2) Verify cloud service provider's organizational and manpower level stability
	(3) Evaluate whether cloud service provider's charging system is reasonable or not

**Table 11:** Evaluation items of network and data center—service provision basis.

Evaluation criteria	Evaluation item
Network and data center—service provision basis (4 items)	(1) Whether a computer system is secured to provide cloud service
	(2) Whether the hardware resource such as servers, storages, and networks is secured to provide cloud service
	(3) Whether the infrastructure is checked with a monitoring tool, software, and so forth, to provide cloud service
	(4) Whether the technical support manpower is secured to consistently provide cloud service

**Table 12:** Evaluation items of network and data center—security.

Evaluation criteria	Evaluation item
Network and data center—security (7 items)	(1) Whether or not physical security measures are established and carried out to protect cloud service user
	(2) Whether the access control to the possessed computer system is carried out or not
	(3) Whether or not activities (firewall, IDS, etc.) are carried out to prevent security incident of cloud service
	(4) Whether cloud service's vulnerabilities (virus, etc.) are periodically checked or not
	(5) Whether or not the data encryption is supported to protect the transmitted information between servers and clients
	(6) Whether or not the security items for network, mail, web, and server security (network, mail, web, server, and terminal and operation management) are periodically checked for cloud service security
	(7) Whether or not the provider carries out periodic activities for the operation management such as periodic reporting, server room's security operation management procedure, other management and operation method regulation, updating procedure, and so forth, for cloud service security

**Table 13:** Evaluation items of service continuity.

Evaluation criteria	Evaluation item
Service continuity (4 items)	(1) Whether or not a plan responding to the service use expansion is established to keep QoS of cloud service
	(2) Whether or not a process (fault details notice, etc.) or measure is established or carried out to recover failures such as service interruption
	(3) Whether or not an internal technical and administrative process is secured to keep performance of cloud service
	(4) Whether or not backup, sync, and recovery measures are established and carried out to consistently provide cloud service

**Table 14:** Evaluation items of customer support.

Evaluation criteria	Evaluation item
Customer support (5 items)	(1) Whether or not a standard installation plan, service construction plan, and service implementation plan are established and carried out to support users
	(2) Whether or not the user education on the service (environment, how to use applications, access method, service scope, content, etc.) is carried out to effectively use cloud service
	(3) Whether or not the manpower and organization are secured for after-service customer support and the follow-up service is carried out
	(4) Whether or not activities (service satisfaction evaluation, service level agreement, etc.) are carried out to keep and assure service satisfaction of users
	(5) Whether or not a compensation plan for users (compensation rule, insurance or not) is prepared and carried out when there is a damage such as service interruption

**Table 15:** Comparison of security among Korea cloud service certification system, ISO 27001, ISMS, and PIMS.

Division	Controlled item	Information protection management system		
		ISO 27001	ISMS	PIMS
Korea cloud service certification system	Availability	—	—	—
	Expandability	—	—	—
	Performance	—	—	—
	Data management	(i) Asset management (ii) Communication and operation management	(i) Information asset classification (ii) Operation management	(i) Personal information classification (ii) Technical protective measure
	Security	(i) All items	(i) All items	(i) All items excluding requirement based on lifecycle
	Service continuity	(i) System transfer (ii) Development and repair/maintenance	(i) System development security	—
	Service support	(i) Communication and operation management	(i) Operation management	(i) Technical protective measure

**Table 16:** Improvement method of certification grade.

As is	To be
Cloud service certification (i) More than 70% of the evaluation indexes are satisfied (ii) IaaS: 105 items/SaaS: 85 items Cloud service certification with excellent SLA (i) Additional items (availability above 99.9%, compensation criteria, ISMS, ISO27001, and certification acquisition) are satisfied in addition to the cloud service certification requirements	Grant a grading system for each certification (levels 1~5) (i) Subdivide cloud service certification grade into two grades and simplify the screening/evaluation items and document for each grade (ii) Subdivide the excellent SLA grade into three grades and differentiate the screening/evaluation items for each grade
